# Draft Genome Sequences of Histamine-Producing *Morganella psychrotolerans* Strains

**DOI:** 10.1128/genomeA.01001-16

**Published:** 2016-09-15

**Authors:** Kristin Bjornsdottir-Butler, Maria Sanchez Leon, Ronald A. Benner

**Affiliations:** a FDA, Division of Seafood Science and Technology, Gulf Coast Seafood Laboratory, Dauphin Island, Alabama, USA; b FDA, Division of Public Health Informatics and Analytics, College Park, Maryland, USA

## Abstract

Histamine-producing bacteria are responsible for scombrotoxin (histamine) fish poisoning, a leading cause of fish poisoning in the United States. We report here the first draft genomes of three histamine-producing *Morganella psychrotolerans* strains, isolated from tuna and mahi-mahi.

## GENOME ANNOUNCEMENT

*Morganella psychrotolerans* is a psychrotolerant, histamine-producing bacterium able to produce toxic concentrations of histamine during cold storage of fish. Strains of these bacteria have been isolated from fresh tuna, cold-smoked tuna, and garfish and can produce toxic concentrations of histamine at temperatures as low as 0°C (1). In addition, these bacteria have been implicated in scombrotoxin fish poisoning outbreaks (2, 3). Since rapid chilling to temperatures ≤4.4°C is recommended to control histamine formation in fish products in the United States (4), these bacteria may be of particular concern in reducing the occurrence of illness.

The *Morganella psychrotolerans* strains sequenced in this study were histamine-producing bacteria, isolated from vacuum-packed mahi-mahi (GCSL-Mp3, GCSL-Mp20) obtained from local supermarkets in Alabama, USA, and cold-smoked tuna (DMS-17786^T^) from Denmark. These strains were sequenced to confirm identification and to characterize the histidine decarboxylase gene.

The genomes were sequenced using the Ion PGM sequencer and Ion OneTouch 2 system with 400-bp reads (Life Technologies, Frederick, MD, USA). Briefly, for DNA purification, single colonies were incubated in 5 ml of tryptic soy broth at 20°C with shaking at 200 rpm for 24 h. DNA was extracted with DNeasy blood and tissue kits according to the manufacturer’s instructions (Qiagen, Valencia, CA, USA). DNA concentrations were determined using a Qubit version 2.0 fluorometer with Qubit dsDNA HS assay kits according to the manufacturer’s instructions (Life Technologies). DNA was enzymatically fragmented using Ion Xpress Plus fragment library kits (Life Technologies) and size was determined with E-Gel SizeSelect 2% agarose gels in an E-Gel iBase unit (Life Technologies). The template for the Ion Torrent PGM instrument was prepared with Ion PGM template OT2 400 kits and sequenced with Ion PGM sequencing kits on an Ion 318 V2 chip according to the manufacturer’s instructions (Life Technologies). For each isolate, the genomic sequence single-pass reads were *de novo* assembled using SPAdes software (5) and annotated using the NCBI Prokaryotic Genome Annotation Pipeline (http://www.ncbi.nlm.nih.gov/genome/annotation_prok) (6). Through the annotation process, 3,890 to 4,178 genes were identified for the *Morganella psychrotolerans* isolates. The identification of the isolates and presence of the histidine decarboxylase gene was confirmed in all isolates.

### Accession number(s).

The draft genome sequences of the three *Morganella psychrotolerans* isolates are available in GenBank under the accession numbers LZEW00000000, LZEX00000000, and LZEY00000000.
